# The evolution of cell-free fetal DNA testing: expanded non-invasive prenatal testing and its effect on target populations

**DOI:** 10.3389/fmed.2025.1522680

**Published:** 2025-01-21

**Authors:** Shaozhe Yang, Yanqi He, Jingshang Lv, Rongxiang Li, Xiuhong Fu

**Affiliations:** Henan Key Laboratory of Fertility Protection and Aristogenesis, Luohe Central Hospital, Luohe, China

**Keywords:** cell-free fetal DNA, prenatal screening, expanded non-invasive prenatal testing, copy number variations, chromosome aneuploidies

## Abstract

**Purpose:**

To evaluate the clinical performance of expanded non-invasive prenatal testing (NIPT-plus) in screening for fetal chromosome aneuploidy and copy number variations (CNVs) among pregnant women with different risk factors to investigate how the target population of cell-free fetal DNA may change in NIPT-plus.

**Methods:**

The clinical data, test results, confirmatory invasive testing outcomes, and follow-up results of 6,220 pregnant women who underwent NIPT-plus were re-viewed. The performance indicators of the positive predictive value (PPV), positive rate (PR), specificity, and sensitivity in screening for common trisomies, sex chromosomal abnormalities (SCAs), rare autosomal aneuploidies (RAAs), and CNVs were calculated. The PR or PPV of NIPT-plus for screening chromosome aneuploidy and CNVs in women of varying ages, risk factors, and clinical indications were determined.

**Results:**

The PRs of common trisomies, SCAs, RAAs, and CNVs in NIPT-plus were 0.71, 0.45, 0.32, and 0.59%, respectively, with 100% sensitivity and specificities ranging from 99.69 to 99.87%. The PPVs were 80.95, 30.77, 13.33, and 44.12%, respectively. The high-risk group had higher PRs and PPVs for chromosome aneuploidy, with no significant difference in screening for CNVs. NIPT-plus showed greater PR for aneuploidy in the older age group than in the younger age group, with no significant differences in CNVs screening.

**Conclusion:**

NIPT-plus was able to effectively screen for chromosome aneuploidy and CNVs. The performance of CNVs screening was not significantly different among different risk factors and age groups. The target population for NIPT-plus should include all pregnant women, not just those at high risk.

## 1 Introduction

Chromosomal abnormalities impact the success rate of assisted reproduction and also contribute to early miscarriages, neonatal deaths, and childhood disabilities (1). Chromosomal abnormalities account for 70–80% of spontaneous abortions (2), 15% of newborns with congenital abnormalities (3), and 25% of newborn deaths (4). Genetic factors alone or in combination are responsible for causing eighty percent of birth defects (5). Birth defects significantly impact the survival and quality of life of affected children, causing immense pain and economic burden to both the children and their families, making them a major public health issue worldwide (6). The main chromosome abnormalities that lead to birth defects include common trisomies (trisomy 21/ trisomy 18/ trisomy 13), sex chromosomal abnormalities (SCAs), copy number variants (CNVs), and rare autosomal aneuploidies (RAAs) (7). Among these, common trisomies and SCAs are of particular concern because of their relatively high incidence rates and have become the primary target diseases for prenatal screening (8).

As next-generation sequencing (NGS) and chromosomal microarray analysis (CMA) have become more widespread, the potential harm caused by CNVs has attracted the attention of obstetricians and pregnant women (9). Pathogenic CNVs can lead to microdeletion and microduplication syndromes (MMSs) at any point in pregnancy, regardless of the mother’s age (10). In addition, MMS causes about 12% of unexplained intellectual disabilities, various deformities and developmental delays (5). Among fetuses with abnormal ultrasound structures, 6% were found to have pathogenic CNVs, while 1.6% of fetuses with normal ultrasounds also had pathogenic CNVs (11), which is much higher than the incidence rate of common trisomies in fetuses (12). All pregnant women should be offered fetal CNVs screening, not just those of advanced maternal age (AMA).

Ever since non-invasive prenatal testing (NIPT) was first introduced in 2011, it has quickly become widely used because of its convenience, non-invasive nature, and precision (13, 14). Multiple professional associations from various countries and international professional associations have issued statements and guidelines recommending NIPT as a primary screening method for fetal chromosome aneuploidy (15). NIPT has a high positive predictive value (PPV) and an extremely low false-negative rate (FNR) in screening for common trisomies (16, 17). Compared to NIPT, expanded noninvasive prenatal testing (NIPT-plus) increased sequencing depth and optimized bioinformatics algorithms, enabling the screening of CNVs (10, 18).

Although multiple studies have demonstrated the reliability of NIPT-plus screening for pathogenic CNVs (19–23), recent research findings have also highlighted the following issues associated with the large-scale application of NIPT-plus: (1) NIPT-plus has a lower PPV and a higher FPR when screening for CNVs than do common trisomies (20, 24, 25); (2) the effectiveness of NIPT-plus in screening for CNVs is influenced by the size of the CNVs (19, 21, 25, 26); (3) NIPT-plus may detect CNVs with unclear pathogenicity, which could complicate genetic counseling (21); (4) pregnant women show a low willingness for subsequent prenatal diagnosis after screening positive for CNVs (27). These issues have sparked debates over the expansion of target diseases for cffDNA testing (26, 28). Neither the American College of Obstetrics and Gynecology nor the European Society of Human Genetics recommend screening for fetal MMSs via cell-free fetal DNA (cffDNA) testing (29). More testing data and follow-up results are needed to support the screening effectiveness of NIPT-plus. Additionally, the shift in the target population for cffDNA testing as NIPT evolves into NIPT-plus needs to be closely monitored. Previous reports have shown that NIPT has a greater PPV for pregnant women in high-risk group, such as AMA (≥ 35 years), abnormal maternal serum screening (AMSS, intermediate risk: 1/1,000 ≤ T21 ≤ 1/270, 1/1,000 ≤ T18 ≤ 1/350 and high risk: T21 ≥ 1/270, T18 ≥ 1/350), ultrasonic anomalies (UA), and previous fetus/child with abnormalities (PFA), than for low-risk pregnant women (without high-risk factors) (30). Therefore, NIPT is more commonly recommended to high-risk pregnant women. There is a significant difference between the populations affected by CNVs and those with chromosome aneuploidy (10), indicating the need for more research to support the changes in the target population of NIPT-plus.

This research reviewed the findings of 6,220 NIPT-plus tests and their follow-up results. A comparison of the results of chromosome aneuploidy and CNVs testing for different risk factors and age groups of pregnant women highlighted the effectiveness of NIPT-plus testing and the shift in the target population for cffDNA testing.

## 2 Methods and materials

### 2.1 Subjects

**FIGURE 1:**
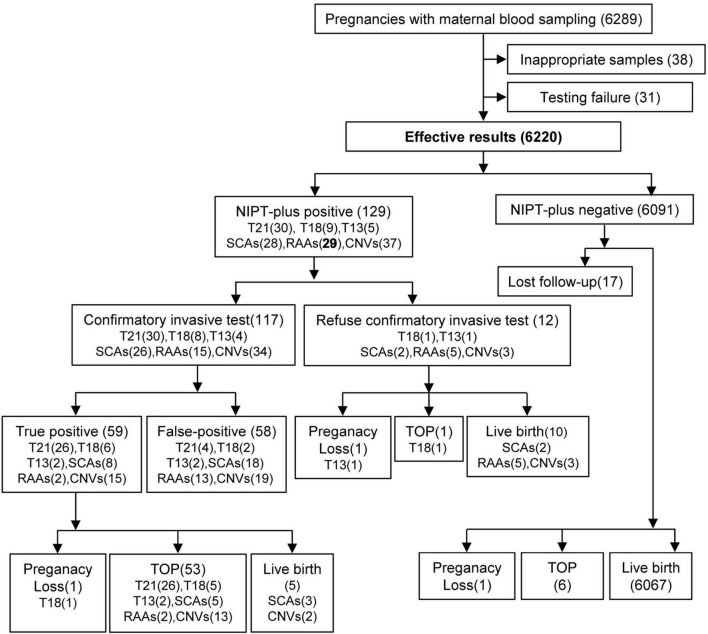
Flowchart of NIPT-plus and outcomes. T21, trisomy 21; T18, trisomy 18; T13, trisomy 13; SCAs, sex chromosome aneuploidies; RAAs, rare autosomal aneuploidies; CNVs, copy number variants; TOP, termination of pregnancy.

This was a retrospective study involving 6,220 pregnant women who underwent NIPT-plus testing at Luohe Central Hospital in China from January 2019 to December 2023. Among the initial 6,289 participants, 38 were excluded because of missing clinical data, and 31 were excluded because of two consecutive test failures. The involvement of pregnant women in this study, along with the results of NIPT-plus testing, confirmatory invasive testing, pregnancy outcomes, and follow-up details, is detailed in Figure 1. The pregnant women who participated in this study were those who had registered for blood collection at Luohe Central Hospital, a prenatal diagnostic institution, as well as seven partner prenatal screening institutions. The clinical data of the research subjects, including their names, ages, heights, weights, gestational ages (GA), gravidities, parities, obstetric histories, genetic disease family histories, parental chromosomal examination results, prenatal serum screening results, prenatal ultrasound screening results, NIPT-plus results, confirmatory invasive testing results, pregnancy follow-up results, modes of delivery, and newborn follow-up results, were collected.

According to the guidelines of the National Health Commission of China, we do not perform NIPT-plus testing for the following six types of pregnant women: (1) those with a GA < 12^+0^ weeks; (2) those or the biological father of the fetus with confirmed chromosomal abnormalities; (3) those who received allogeneic blood transfusion, transplant surgery, or allogeneic cell therapy within the past year; (4) those with fetal ultrasound findings suggesting structural abnormalities; (5) those with family history of fetal genetic diseases; and (6) pregnant women with malignant tumors during pregnancy. All pregnant women must receive genetic counseling; be informed about the testing methods, target diseases, and potential for false-positives and false-negatives; and sign a written consent form. This study followed strict privacy protection regulations and was approved by the Medical Ethics Committee of Luohe Central Hospital (NO. MEC-2018-076).

### 2.2 NIPT-plus

The NIPT-plus test was conducted following a previously reported procedure (31). A Streck Cell-Free DNA BCT blood collection tube (Streck, La Vista, Nebraska, USA) was used to collect 10 mL of peripheral blood from pregnant women, and plasma separation was completed within 96 h after blood collection. The plasma separation technique utilizes the “two-step centrifugation method,” where the collected blood sample is first centrifuged at 1,600 × *g* in 4°C for 10 min, followed by centrifugation at 16,000 × *g* in 4°C for 10 min in order to eliminate any remaining blood cells. The plasma cell-free DNA extraction kit, fetal chromosome aneuploidy testing kit, NGS library construction, and DNA purification kit produced by Berry Genomics in China were used for the extraction, library construction, and library quantification of cffDNA. By employing the Illumina NextSeq CN500 sequencer for high-throughput gene sequencing, the data quality control criteria included cffDNA% ≥ 4% and Uniq Reads ≥ 10 million. The gene sequence obtained from the sequencer was mapped to the human reference genome sequence GRCh37 (hg19) in order to identify chromosomal abnormalities. The evaluation of chromosome aneuploidy was based on Z values: | Z| ≥ 3 indicated a high risk of chromosome aneuploidy, whereas | Z| < 3 indicated a low risk. Hidden Markov models (HMMs) were utilized for the detection of CNVs (32).

### 2.3 Confirmatory invasive testing

All pregnant women with a positive NIPT-plus result were recommended to undergo confirmatory invasive testing. For pregnant women with high risk of fetal chromosomal aneuploidy, the recommended confirmatory invasive testing options were amniocentesis and amniotic fluid chromosome karyotype analysis. For pregnant women with high risk of fetal chromosomal CNVs, the recommended confirmatory invasive testing approach included amniocentesis, amniotic fluid karyotyping analysis, and amniotic fluid CNV-sequencing (CNV-Seq). By utilizing fluorescent quantitative PCR (QF-PCR), a swift diagnosis of common chromosome aneuploidies (13/18/21/XY) was performed on every amniotic fluid sample to exclude any maternal cell contamination present in the amniotic fluid.

Amniocentesis: After 18 weeks of pregnancy, approximately 20 mL of amniotic fluid was extracted from the pregnant woman under ultrasound guidance. For amniotic fluid karyotyping analysis, the amniotic fluid was inoculated on the same day as the puncture surgery. For CNV-Seq, the amniotic fluid was stored at 4°C, and genomic DNA extraction was performed within 48 h.

Chromosome karyotype analysis: Following centrifugation of approximately 7 mL of amniotic fluid, it was cultured in amniotic fluid culture medium (Da Hui Bioscience, Guangzhou, China or BI, Beit Haemek, Israel). After cultivation, collection, banding, and karyotype analysis, the G-banding karyotype was scanned and analyzed via a fully automated chromosome karyotyping system (Carl Zeiss, Jena, Germany). For each sample, a minimum of 5 different karyotypes were analyzed, with a count of at least 20 karyotypes. The karyotype was defined according to the International System for Human Cytogenomic Nomenclature 2019 (ISCN 2019) standard.

CNV-seq: After extracting genomic DNA from amniotic fluid via the QIAamp DNA Mini Kit (Qiagen, NY, USA), quantification was performed via the Invitrogen Qubit™ 5.0 (Thermo Fisher Scientific, MA, USA). The CNVs detection kits, NGS library construction kits, and DNA purification kits from Berry Genomics in Beijing, China, were used for CNVs sequencing library construction, purification, quality control, and other related procedures. NGS was performed via the Illumina NextSeq CN500 sequencer, with a data quality control standard of at least 8 million unique reads. The Xromate^®^ analysis system (Berry Genomics, Beijing, China) was used to analyze the sequencing data, with the human reference genome sequence GRCh37 (hg19) being employed. In accordance with the guidelines set forth by the American College of Medical Genetics (ACMG), the pathogenicity of CNVs was assessed by referencing public databases (ClinGen, DECIPHER, DGV, OMIM, NCBI, UCSC) to determine their classification as benign, likely benign, variants of uncertain significance (VUS), likely pathogenic, or pathogenic (33).

### 2.4 Clinical follow-up assessment

Pregnant women with a positive confirmatory invasive test result could decide on their own whether to undergo termination of pregnancy (TOP) after genetic counseling. For pregnant women with a negative NIPT-plus result or negative confirmatory invasive test results, regular prenatal care and ultrasound examinations were recommended.

Phone follow-ups were conducted for all pregnant women who had undergone NIPT-plus testing, with live birth mothers receiving at least two follow-ups—one before delivery and another three months postpartum. The follow-up before delivery included prenatal check-up information, whereas the follow-up after delivery included modes of delivery, newborn outcomes, newborn physical examination, and developmental details. The outcomes of pregnancy could be categorized into four groups: pregnancy loss, TOP, live birth, and loss to follow-up.

### 2.5 Statistical analysis

Data analysis was conducted via SPSS 25.0 software (SPSS Inc., Chicago, IL, USA). Descriptive statistics are presented as the means and min–max, whereas categorical data are presented as the rates. On the basis of the results of NIPT-plus and confirmatory invasive testing, the PPV, positive rate (PR), sensitivity and specificity of each target disease could be calculated to evaluate the performance of NIPT-plus. When two or more groups were compared, a chi-square test was used, with a significance level of *p* < 0.05 indicating a statistically significant difference. The use of amniotic fluid karyotype results or CNV-Seq results is the gold standard for fetal chromosomal diagnosis. Pregnant women who refused confirmatory invasive testing or were lost follow-up were excluded. The sensitivity, PPV, and specificity are calculated as follows: PPV = [TP/ (TP+FP)] × 100%, sensitivity = [TP/ (TP+FN)] × 100%, and specificity = [TN/ (TN+FP)] × 100%, where TP: true positive; FP: false-positive; TN: true negative; FN: false-negative.

## 3 Results

### 3.1 Demographic characteristics of pregnant women underwent NIPT-plus

The demographic traits of the pregnant women involved in this study are summarized in Table 1. The pregnant women had an average age of 30.15 years, ranging from 18 to 43 years. A total of 61.99% of the samples were from prenatal diagnosis center, whereas 38.01% were from partner prenatal screening institutions. The average GA of pregnant women during blood sampling was 18.65 weeks, ranging from 12 to 31 weeks. Among them, 7.59% were at 12–13 weeks, 79.90% were at 14–27 weeks, and 12.51% were at 28 weeks or more. Among the 6,220 pregnant women included in the study, 3.71% were pregnant with twins, with no pregnant women carrying triplets or more included in this study.

**TABLE 1:** Demographic characteristics of women underwent NIPT-Plus.

Characteristic	Total population (*n* = 6,220)
**Maternal age (years)**	
Mean, min–max	30.15, 18–43
< 30	1,822 (29.29%)
30–34	2,363 (37.99%)
≥ 35	2,035 (32.72%)
**Hospitals for blood drawn**	
Prenatal diagnosis center	3,856 (61.99%)
Partner prenatal screening institutions-1	1,417 (22.78%)
Partner prenatal screening institutions-2	305 (4.90%)
Partner prenatal screening institutions-3	257 (4.13%)
Partner prenatal screening institutions-4	115 (1.85%)
Partner prenatal screening institutions-5	108 (1.74%)
Partner prenatal screening institutions-6	98 (1.58%)
Partner prenatal screening institutions-7	64 (1.03%)
**Gestational age (weeks)**	
Mean, min–max	18.65, 12–31
1st trimester (12–13 weeks)	472 (7.59%)
2nd trimester (14–27 weeks)	4,970 (79.90%)
3rd trimester (?28 weeks)	778 (12.51%)
**Number of fetuses**	
1	5,989 (96.29%)
2	231 (3.71%)
> 2	0 (0.00%)
**Detection indication**	
AMA	2,035 (32.72%)
AMSS	1,402 (22.54%)
UA	241 (3.87%)
PFA	68 (1.09%)
Twin pregnancy	215 (3.46%)
IVF	240 (3.86%)
Routine screening	2,019 (32.46%)

AMA, advanced maternal age; AMSS, abnormal maternal serum screening; UA, ultrasonic anomalies; PFA, previous fetus/child with abnormalities; IVF, *in vitro* fertilization.

According to the information registered during blood collection, all pregnant women were classified into seven categories for testing indications: AMA, AMSS, UA, PFA, twin pregnancy, *in vitro* fertilization (IVF), and routine screening. The highest proportion was AMA (2,035, 32.72%), followed by routine screening (2,019, 32.46%), AMSS (1,402, 22.54%), UA (241, 3.87%), IVF (240, 3.86%), twin pregnancy (215, 3.46%), and PFA (68, 1.09%).

### 3.2 Performance of NIPT-plus for screening chromosome aneuploidy and CNVs

In total, 129 cases with a high risk of chromosomal abnormalities were detected among 6,220 pregnant women who received NIPT-plus testing, leading to a composite PR of 2.07%. Overall, 117 pregnant women who tested positive on NIPT- plus underwent confirmatory invasive testing, resulting in 59 cases being confirmed as true positives. The composite PPV was 50.43%, with a composite sensitivity of 100% and a composite specificity of 99.06% (Table 2).

**TABLE 2:** Performance of NIPT-plus in screening for fetal chromosome abnormalities.

Abnormal type	NIPT-plus positive		Confirmatory invasive testing		True positive	False positive	PPV	Sensitivity	Specificity
	***n***	**%**	***n***	**%**	***n***	***n***	**%**	**%**	**%**
Common trisomies	44	0.71	42	95.45	34	8	80.95	100.00	99.87
T21	30	0.48	30	100.00	26	4	86.67	100.00	99.94
T18	9	0.14	8	88.89	6	2	75.00	100.00	99.97
T13	5	0.08	4	80.00	2	2	50.00	100.00	99.97
SCAs	28	0.45	26	92.86	8	18	30.77	100.00	99.71
45,X	10	0.16	9	90.00	2	7	22.22	100.00	99.89
47,XXX	5	0.08	4	80.00	2	2	50.00	100.00	99.97
47,XXY	9	0.14	9	100.00	3	6	33.33	100.00	99.90
47,XYY	4	0.06	4	100.00	1	3	25.00	100.00	99.95
RAAs	20	0.32	15	75.00	2	13	13.33	100.00	99.79
CNVs	37	0.59	34	91.89	15	19	44.12	100.00	99.69
Total	129	2.07	117	90.70	59	58	50.43	100.00	99.06

PPV, positive predictive value; T21, trisomy 21; T18, trisomy 18; T13, trisomy 13; SCAs, sex chromosome aneuploidies; RAAs, rare autosomal aneuploidies; CNVs, copy number variants.

In total, 44 cases (0.71%) were identified as high risk for common trisomies through NIPT-plus, with 30 cases of T21 (0.48%), 9 cases of T18 (0.14%), and 5 cases of T13 (0.08%). Except for one T18 high-risk pregnant woman who chose TOP due to fetal ultrasound abnormalities and one T13 high-risk pregnant woman who experienced a miscarriage, the remaining 42 cases all underwent confirmatory invasive testing, with 95.45% receiving confirmatory invasive testing. In total, 34 true positive cases were found, including 26 cases of T21, 6 cases of T18, and 2 cases of T13. The PPV for common trisomies is 80.95%. Specifically, the PPVs for T21, T18, and T13 were 86.67, 75.00, and 50.00%, respectively.

The PR for SCAs in NIPT-plus screening was 0.45% (28 cases), with 10 (0.16%) cases of 45,X, 5 (0.08%) cases of 47,XXX, 9 (0.14%) cases of 47,XXY, and 4 (0.06%) cases of 47,XYY high risk. Among the 26 high-risk pregnant women with SCAs, confirmatory invasive testing was conducted, resulting in 8 true positive cases. These included 2 cases of 45,X, 2 cases of 47,XXX, 3 cases of 47,XXY, and 1 case of 47,XYY. The PPVs for these four types of SCAs were 22.22, 50.00, 33.33, and 25.00%, respectively. The combined PPV for SCAs was 30.77%.

Among the 20 high-risk pregnant women identified by NIPT-plus for RAAs (0.32%), 15 (75.00%) underwent confirmatory invasive testing. Among them, 2 fetuses were confirmed to have chromosomal aneuploidies, with fetal chromosomal karyotypes of 47,XN,+20[44]/,46,XN [54] and 47,XN,+10[10]/46,XN[37]. The overall PPV for RAAs was only 13.33%.

Overall, 37 cases (0.59%) of high-risk CNVs were detected in pregnant women through NIPT-plus testing. Among these pregnant women, 34 (91.89%) underwent fetal amniotic fluid chromosomal karyotyping analysis and CNV-Seq testing, leading to the confirmation of 15 pathogenic CNVs. The comprehensive PPV of CNVs was 44.12%.

### 3.3 Performance of NIPT-plus in screening chromosomal abnormalities for various risk levels and detection indications

Pregnant women who have at least one of the following risk factors—AMA, AMSS, UA, or PFA—were classified into the high-risk group. Pregnant women without these risk factors were classified into the low-risk group. A comparison of the high-risk and low-risk groups in screening for chromosome aneuploidy and CNVs is shown in Table 3. The results indicate differences in the PR and PPV. In screening for chromosome aneuploidy, the PPV and PR were greater in the high-risk group than in the low-risk group (PR: 1.76 vs. 1.05%, *p* = 0.023; PPV: 61.67 vs. 30.43%, *p* = 0.011). In the screening for CNVs, the PR and PPV of the high-risk group were lower than those of the low-risk group, however, the distinction did not reach statistical significance (PR: 0.59 vs. 0.61%, *p* = 0.924; PPV: 38.10 vs. 53.85%, *p* = 0.484). When the results of the chromosome aneuploidy and CNVs screening were combined, the high-risk group had greater PRs and PPVs than did the low-risk group, while there was no statistically significant difference observed (PR: 2.35 vs. 1.66%, *p* = 0.061; PPV: 55.56 vs. 38.89%, *p* = 0.096).

**TABLE 3:** Comparison of NIPT-plus performance between the low-risk group and the high-risk group.

Performance	Aneuploidy			CNVs			Aneuploidy and CNVs		
	High risk	Low risk	*P*-value	High risk	Low risk	*P*-value	High risk	Low risk	*P*-value
Test positive (*n*)	66	26	NA	22	15	NA	88	41	NA
Positive rate (%)	1.76	1.05	0.023	0.59	0.61	0.924	2.35	1.66	0.061
Confirmatory invasive testing (*n*)	60	23	NA	21	13	NA	81	36	NA
True positive (*n*)	37	7	NA	8	7	NA	45	14	NA
False-positive (*n*)	23	16	NA	13	6	NA	36	22	NA
Positive predictive value (%)	61.67	30.43	0.011	38.10	53.85	0.484	55.56	38.89	0.096

PPV, positive predictive value; CNVs, copy number variants; NA, not applicable.

In screening for chromosome aneuploidy, the order of testing indications from highest to lowest PR was as follows: PFA > UA > AMSS > AMA > IVF > routine screening > twin pregnancy. Moreover, in screening for CNVs, the corresponding order was IVF = UA > routine screening = AMA > AMSS > twin pregnancy > PFA, as shown in Table 4.

**TABLE 4:** Comparison of performance of NIPT-Plus across various detection indications.

Detection indication	Test number	NIPT-plus positive result					
		Aneuploidy		CNVs		Aneuploidy and CNVs	
		*n*	%	*N*	%	*N*	%
AMA	2,035	29	1.43	12	0.59	41	2.01
AMSS	1,402	26	1.85	8	0.57	34	2.43
UA	241	8	3.32	2	0.83	10	4.15
PFA	68	3	4.41	0	0.00	3	4.41
Twin pregnancy	215	2	0.93	1	0.47	3	1.40
IVF	240	3	1.25	2	0.83	5	2.08
Routine screening	2,019	21	1.04	12	0.59	33	1.63

AMA, advanced maternal age; AMSS, abnormal maternal serum screening; UA, ultrasonic anomalies; PFA, previous fetus/child with abnormalities; IVF, *in vitro* fertilization; CNVs, copy number variants.

A comparison of PRs in different age groups of pregnant women for NIPT-plus revealed that the PRs for screening for chromosome aneuploidy increased with age, with PRs of 0.99, 1.40, and 2.01% for the < 30 years, 30–34 years, and ≥ 35 years groups, respectively. The differences among the three groups were statistically significant (*p* = 0.028). When CNVs were screened, the PRs for the < 30 years, 30–34 years, and ≥ 35 years groups were 0.55, 0.63, and 0.59%, respectively, there were no statistically significant differences between the groups, with a *p*-value of 0.937, as shown in Table 5.

**TABLE 5:** Performance comparison of NIPT-plus among pregnant women of different ages.

Maternal age (years)	Test number	NIPT-plus positive result					
		Aneuploidy		CNVs		Aneuploidy and CNVs	
		*N*	%	*N*	%	*N*	%
< 30	1,822	18	0.99	10	0.55	28	1.54
30–34	2,363	33	1.40	15	0.63	48	2.03
≥ 35	2,035	41	2.01	12	0.59	53	2.60
*P*-value	NA	NA	0.028	NA	0.937	NA	0.066

CNVs, copy number variants.

### 3.4 Pregnancy outcomes of NIPT-plus-positive pregnant women and follow-up data

Among the pregnant women who received NIPT-plus screening, all those with positive screening results were successfully followed up. However, 17 pregnant women with low-risk screening results were lost to follow-up, resulting in a loss rate of 0.27% (17/6,220). For 59 pregnant women, confirmatory invasive testing results confirmed true positive results, with pregnancy outcomes outlined in Table 6. Among the 34 cases of true positive common trisomies, only 1 case of T18 resulted in spontaneous miscarriage before TOP was chosen, while the remaining 33 cases opted for TOP. Among the 8 cases of true positive SCAs, 5 opted for TOP, whereas 3 continued with the pregnancy and gave birth to babies (including 1 case of 45,X, 1 case of 47,XXX, and 1 case of 47,XXY). No abnormalities were observed in the newborns, and chromosomal analysis was not conducted. The continuation rate of pregnancies with SCAs was 37.50% (3/8). Both pregnant women with 2 confirmed cases of RAAs opted for TOP. Among the 15 pregnant women with true positive CNVs, 13 chose TOP, while the other 2 pregnant women chose to continue their pregnancies and deliver. One case showed Dup (22) (q11.21) on NIPT-plus, while the amniotic fluid CNV-seq revealed Dup (15) (q13.3). In another case, both the NIPT-plus and amniotic fluid CNV-seq results indicated 22q11 deletion syndrome. The TOP rate for common trisomies was 100% (34 out of 34), followed by RAAs at 100% (2 out of 2), CNVs at 86.67% (13 out of 15), and SCAs at 62.50% (5 out of 8).

**TABLE 6:** Pregnancy outcomes of true positive cases in NIPT-plus.

Chromosomal abnormality	True positive	Outcome of true positive cases			
		Pregnancy loss	Termination of pregnancy	Live birth	Loss follow-up
Common trisomies	34	1	33	0	0
T21	26	0	26	0	0
T18	6	1	5	0	0
T13	2	0	2	0	0
SCAs	8	0	5	3	0
45,X	2	0	1	1	0
47,XXX	2	0	1	1	0
47,XXY	3	0	2	1	0
47,XYY	1	0	1	0	0
RAAs	2	0	2	0	0
CNVs	15	0	13	2	0
Total	59	1	53	5	0

T21, trisomy 21; T18, trisomy 18; T13, trisomy 13; SCAs, sex chromosome aneuploidies; RAAs, rare autosomal aneuploidies; CNVs, copy number variants.

The clinical outcomes of pregnant women with NIPT-plus-positive results but who refused confirmatory invasive testing are shown in Table 7. One pregnant woman with a positive T13 NIPT-plus result had an ultrasound that revealed fetal structural abnormalities, leading to a subsequent miscarriage. Another pregnant woman with a positive T18 NIPT-plus result chose to undergo TOP because of fetal ultrasound abnormalities. Two pregnant women with NIPT-positive SCAs (one with 45,X and one with 47,XXX) declined confirmatory invasive testing and gave birth, with no abnormalities observed in the newborns. Five pregnant women with positive RAA NIPT-plus results (T8, T22, T5, T7, and T9) and three pregnant women with positive CNVs NIPT-plus results [dup (4) (q12-q13.1); size: 5.5 M, Del (7) (q1.12-q2.3); size: 5.8 M, Dup (9) (q12-q21.11); size: 6.5 M] refused confirmatory invasive testing and chose to give birth. The newborns showed no abnormalities.

**TABLE 7:** Prenatal ultrasound and follow-up outcomes of NIPT-plus positive cases refused confirmatory invasive testing.

Case	NIPT-Plus result	Prenatal ultrasound	Pregnancy outcome	Fetal outcome
1	Trisomy 18	Choroid plexus cysts and Clenched hands	TOP	NA
2	Trisomy 13	Hyperamniotic fluid and Ventricular septal defect	Pregnancy loss	NA
3	45,X	Normal	Live birth	Normal
4	47,XXX	Normal	Live birth	Normal
5	Trisomy 8	Normal	Live birth	Normal
6	Trisomy 22	Normal	Live birth	Normal
7	Trisomy 5	Normal	Live birth	Normal
8	Trisomy 7	Normal	Live birth	Normal
9	Trisomy 9	Normal	Live birth	Normal
10	CNV: Dup (4) (q12-q13.1);size: 5.5 M	Normal	Live birth	Normal
11	CNV: Del (7) (q1.12-q2.3);size: 5.8 M	Normal	Live birth	Normal
12	CNV: Dup (9) (q12-q21.11);size: 6.5 M	Normal	Live birth	Normal

Del, deletion; Dup, duplication; CNV, copy number variant; NA, not applicable.

Among the 6,074 pregnant women who tested negative for NIPT-plus and had successful follow-ups, 6,067 had live births, and no newborns displaying chromosome abnormalities during the follow-up. One pregnant woman experienced spontaneous abortion, four pregnant women opted for TOP due to various reasons for fetal ultrasound abnormalities, and two pregnant women chose TOP despite no abnormalities in the fetus. All pregnant women who underwent NIPT-plus testing in this study had their basic information and test results documented in the Supplementary materials.

## 4 Discussion

Although there is still debate among scholars about the effectiveness of NIPT-plus, published research results have also indicated the significant potential of cffDNA testing, from screening for chromosome aneuploidy to detecting CNVs and even indicating maternal malignancies (34). Discussion on NIPT-plus should not be limited to its testing performance but should also focus on standard operating procedures, reasonable target diseases, and a suitable target population. These factors are crucial for the widespread application of this technology. This study, which is based on clinical data, evaluated the detection performance of NIPT-plus and compared the differences in the detection efficiency of NIPT-plus for different risk factors, testing indications, and age groups of pregnant women with respect to chromosome aneuploidy and CNVs.

This study demonstrated that NIPT-plus had a sensitivity of 100% for Common trisomies, with a specificity exceeding 99%, which is consistent with previous research (35). In this study, the PPVs for T21, T18, and T13 were 86.67, 75.00, and 50.00%, respectively. The composite PPV for T21/T18/T13 was 80.95%, which is consistent with the reported PPV ranges for T21, T18, and T13 in the literature (71–100, 48–85, and 11–54%, respectively) (19, 22, 36, 37). Like NIPT, NIPT-plus could effectively screen for T21, T18, and T13, which are the most important target diseases for cffDNA testing.

Although the sensitivity of NIPT-plus screening for four SCAs in this study was 100% and the specificity was greater than 99%, the PPV for SCAs was lower than the PPV for common trisomies. There is significant variation in the reported PPVs for SCAs in previous research, ranging from 38.46 to 68.00% (27, 30, 37, 38). These discrepancies in PPV could be a result of the different bioinformatics computational methods used to analyze the sequencing data (27), for example, while this study and Porreco et al.’s (39) research used z-scores to assess SCAs, Hooks et al. (40) developed a chromosome-selective method for evaluating SCAs. Mazloom et al. (41), on the other hand, created a classification algorithm for detecting SCAs. However, in many studies, authors may not have focused on bioinformatics algorithms for assessing the risk of SCAs. In this study, the PPVs for 45,X, 47,XXX, 47,XXY, 47,XYY, and the combination of all four were 22.22, 50.00, 33.33, 25.00, and 30.77%, respectively. The PPV of 45,X in the four SCAs was the lowest, possibly due to (1) the presence of more homologous sequences between the X and the Y chromosomes, leading to sequencing errors with shorter reads in NIPT-plus (42); (2) false-positives caused by placental confined mosaicism, disappearance of one X monosomy in a twin, or maternal X monosomy mosaic (43). Some studies indicate that the low PPV of 45,X might be attributed to the X chromosome’s high GC content, resulting in low amplification efficiency (22). However, in this study involving cffDNA testing, there was no need for PCR amplification of cffDNA libraries before sequencing (31), thus ruling out this factor. The lower PPV of SCAs and the relatively mild clinical symptoms of certain SCAs (43), such as 45,X and 47,XXX, should alert genetic counselors to avoid TOP without confirmatory invasive testing.

The inclusion of RAAs as a target disease for cffDNA screening has always been controversial (44) because of its low PPV and low incidence rate (22, 23), often resulting in early pregnancy loss (45). In this study, out of 6,220 cases of NIPT-plus, a total of 20 high-risk cases of RAAs were identified. Among them, 15 pregnant women underwent confirmatory invasive testing, resulting in the diagnosis of 2 fetuses with mosaic RAAs. The composite PPV was 13.33%, which was higher than the range reported in the literature, possibly due to data bias caused by the small sample size. The high FPR of RAAs, reaching 86.67% (13/15), could potentially be attributed to confined placental mosaicism (CPM) (46). The cffDNA identified through NIPT-plus in maternal blood is thought to primarily come from the apoptosis of placental trophoblast cells and may not entirely reflect the fetus (5). This type of chromosomal abnormality, which occurs only in the placenta and not in the fetus, is known as confined placental mosaicism (CPM), with a reported incidence rate of approximately 1–2% (47). In early pregnancy, RAAs can be fatal, with only fetuses that undergo “trisomy rescue” being able to survive (45). In such cases, cffDNA testing may indicate a high risk of trisomy, whereas fetal tissues present no trisomy cells. Additionally, the “trisomy rescue” of fetuses with RAAs may lead to fetal chromosomal uniparental disomy (UPD) (44). When screening for RAAs, it is important to note that, during confirmatory invasive testing, (1) RAAs may involve mosaicism or UPD. Therefore, when a confirmatory invasive testing method is selected, it is crucial to choose a method that can detect mosaicism, such as CMA or fluorescence *in situ* hybridization (FISH), as well as methods that can detect UPD, such as CMA. The fetal chromosomal karyotype result alone may not accurately reflect the occurrence of RAAs involving mosaicism or UPD. (2) Despite the fact that most RAAs have been proven to be false-positives, pregnant women with a positive RAA screening result have a greater risk of developing pregnancy-related disorders. A large number of trisomy cells in the placenta can lead to placental dysfunction, affecting fetal growth (48). CPM is associated with a series of adverse outcomes for both the fetus and the mother, such as preterm birth, multiple congenital anomalies, stillbirth and fetal growth restriction (27). It is recommended that regular ultrasound monitoring be conducted for all pregnant women with positive RAA screening results.

Like RAAs, the effectiveness of NIPT-plus in detecting CNVs is also a subject of debate (49). Studies have reported varying PPVs for NIPT-plus screening for CNVs, ranging from 15 to over 60% (23, 24, 44, 50), which are generally lower than the PPV for common trisomies. In this study, the comprehensive PPV of CNVs was 44.12%. Currently, the use of cffDNA for CNVs screening focuses mainly on screening for pathogenic CNVs and likely pathogenic CNVs (12). The CNVs involved in this study were all pathogenic or likely pathogenic. NIPT-plus is capable of detecting CNVs at the subchromosomal level because of its increased sequencing depth and data volume. However, the sequencing depth required varies significantly across CNVs of different sizes, ranging from 100 kb to 10 Mb. According to the literature, the PPV of CNVs is closely related to their size, with NIPT-plus showing higher screening efficiency for CNVs larger than 10 Mb than for those smaller than 10 Mb (19, 50). Additionally, smaller CNVs are more likely to be benign variants (51). Evaluating the reliability of CNVs detection in NIPT-plus screening should be limited to a specific sequencing depth and a defined range of CNVs sizes. The International Society of Prenatal Diagnosis (ISPD) recommends that, when CNVs are screened via NIPT, the range should be restricted and integrated with clinical practice (52). Notably, karyotype analysis can detect chromosomal abnormalities of only approximately 10 Mb or larger. Using cffDNA to screen for CNVs can overcome the limitations of chromosomal karyotype analysis (20). Additionally, there is no alternative method for screening CNVs other than obtaining fetal tissues through invasive prenatal diagnosis for CMA or CNV-seq testing (12).

In contrast to the increasing incidence of chromosome aneuploidy with increasing maternal age (53), the occurrence rates of MMSs and other CNVs remain unaffected by maternal age (10). Furthermore, there is no evidence suggesting a link between the occurrence of CNVs and abnormal serum screening. In reality, the occurrence of MMSs is more common than chromosome aneuploidy among young pregnant women (54), highlighting the need for attention to screen for CNVs in this population. Previous research has indicated that in the screening of common trisomies and SCAs via NIPT, the high-risk group tends to have a higher PPV than the low-risk group (30). This study focused on exploring the differences among pregnant women of different ages, with different risk factors or different detection indicators in screening for chromosome aneuploidy or CNVs by NIPT-plus. The aim of this study was to investigate whether the traditional “high-risk population for NIPT” is suitable for CNVs screening. The results indicate that the traditional “NIPT high-risk population” or AMA women do not show a higher PR or PPV in screening for CNVs, indicating that the target population for NIPT-plus is clearly different from that for NIPT. Although some organizations, such as the American College of Obstetricians and Gynecologists (ACOG), recommend the use of NIPT for screening for aneuploidy in both high-risk and low-risk pregnant women (55), owing to the relatively high cost of NIPT, it may be more commonly recommended for pregnant women with high-risk factors. The cffDNA testing specifications set by the National Health Commission of China only specify that the “target population” for NIPT includes those with intermediate risk in serum prenatal screening (1/1,000 ≤ T18 ≤ 1/350; 1/1,000 ≤ T21 ≤ 1/270), those with contraindications to invasive prenatal diagnosis, and pregnant women at 20^+6^ weeks or later who have missed the optimal timing for serum screening. As NIPT-plus becomes more common, the target population for NIPT-plus should be expanded further, especially with a focus on young pregnant women (12).

There are several limitations in this study: (1) the sample size was not large, leading to potential data bias in some of the detection indicators, especially for chromosome abnormalities with low PPVs such as SCAs and RAAs, which should be noted; (2) owing to limitations of the analysis software, this study only focused on pathogenic CNVs or likely pathogenic CNVs, without considering other types of CNVs; (3) this study excluded NIPT-plus screening positive cases who refused confirmatory invasive testing and those who were lost to follow-up, which may have impacted the accuracy of the results and consequently influenced the interpretations of this research; (4) it is considered premature in this study to assess whether newborns have SCAs or CNVs on the basis of the three-month postpartum follow-up results. While common trisomies exhibit noticeable chromosomal disorder symptoms at birth, signs of pathogenic CNVs or SCAs may not become apparent until childhood (37).

## 5 Conclusion

While NIPT-plus can successfully screen for chromosome aneuploidy and CNVs, its ability to detect SCAs and RAAs is limited by a lower PPV. The efficacy of NIPT-plus screening for CNVs is not dependent on whether pregnant women have high-risk factors or their age; therefore, the target population for NIPT-plus screening should be expanded to include all pregnant women.

## Data Availability

The original contributions presented in this study are included in this article/Supplementary material, further inquiries can be directed to the corresponding author.
